# Relations of non-motor symptoms and dopamine transporter binding in REM sleep behavior disorder

**DOI:** 10.1038/s41598-019-51710-y

**Published:** 2019-10-29

**Authors:** Petr Dušek, Veronika Lorenzo y Losada Ibarburu, Ondrej Bezdicek, Irene Dall’antonia, Simona Dostálová, Petra Kovalská, Radim Krupička, Jiří Nepožitek, Tomáš Nikolai, Michal Novotný, Pavla Peřinová, Jan Rusz, Tereza Serranová, Tereza Tykalová, Olga Ulmanová, Zuzana Mecková, Václav Ptáčník, Jiří Trnka, David Zogala, Evžen Růžička, Karel Šonka

**Affiliations:** 1 Department of Neurology and Center of Clinical Neuroscience, First Faculty of Medicine, Charles University and General University Hospital in Prague, Prague, Czech Republic; 2 Institute of Nuclear Medicine, First Faculty of Medicine, Charles University and General University Hospital in Prague, Prague, Czech Republic; 3 Department of Biomedical Informatics, Faculty of Biomedical Engineering, Czech Technical University in Prague, Kladno, Czech Republic; 4 Department of Circuit Theory, Faculty of Electrical Engineering, Czech Technical University in Prague, Prague, Czech Republic

**Keywords:** Neurodegeneration, Sleep disorders

## Abstract

The aim of this study was to evaluate associations of motor and non-motor symptoms with dopamine transporter binding in prodromal stage of synucleinopathies. We examined 74 patients with idiopathic REM sleep behavior disorder (RBD), which is a prodromal synucleinopathy, and 39 controls using Movement Disorders Society-Unified Parkinson’s Disease Rating Scale (MDS-UPDRS), Montreal Cognitive Assessment, University of Pennsylvania Smell Identification Test (UPSIT), Farnsworth-Munsell 100 hue test, orthostatic test, Scales for Outcomes in PD-Autonomic, Beck depression inventory-II, State-Trait Anxiety Inventory, and video-polysomnography. Electromyographic muscle activity during REM sleep was quantified according to Sleep Innsbruck-Barcelona criteria. In 65 patients, dopamine transporter single-photon emission computed tomography (DAT-SPECT) imaging was performed, putaminal binding ratio was calculated and scans were classified as normal, borderline, or abnormal. Compared to controls, RBD patients had significantly more severe scores in all examined tests. Patients with abnormal DAT-SPECT had higher MDS-UPDRS motor score (p = 0.006) and higher prevalence of orthostatic hypotension (p = 0.008). Putaminal binding ratio was positively associated with UPSIT score (p = 0.03) and negatively associated with tonic (p = 0.003) and phasic (p = 0.01) muscle activity during REM sleep. These associations likely reflect simultaneous advancement of underlying pathology in substantia nigra and susceptible brainstem and olfactory nuclei in prodromal synucleinopathy.

## Introduction

Idiopathic REM sleep behavioral disorder (RBD) is an early manifestation of neurodegenerative disorders from the synucleinopathy group; 70–90% RBD patients will eventually develop one of the synucleinopathy phenotypes: Parkinson disease (PD), Lewy body dementia (LBD), or multiple system atrophy (MSA)^1,2^. Therefore, RBD patients along with carriers of mutations causing monogenic PD are best populations to study prodromal synucleinopathy^3^. The term prodromal synucleinopathy refers to the stage wherein early symptoms of neurodegeneration are present, but classic clinical diagnosis based on fully evolved parkinsonism or dementia is not yet possible^4^. Previous studies have shown that RBD is frequently associated with high prevalence of several non-motor symptoms such as hyposmia, constipation, orthostasis, anxiety, depression, impaired color vision, and cognitive impairment^5^. These symptoms are likely caused by abnormal alpha-synuclein aggregation in nervous system as was documented in biopsies from colonic mucosa^6^, skin^7^ and salivary glands^8^. In addition, subtle motor symptoms including impairment of speech^9^, oculomotor function^10^ and gait^11^ can be observed before RBD patients reach the clinical threshold for parkinsonism.

It was suggested that alpha-synuclein pathology spreads in the nervous system along predisposed pathways and triggers progressive neurodegeneration in susceptible areas^3^. Gradually progressing degeneration of dopaminergic neurons in substantia nigra (SN) in RBD can be visualized as decreasing tracer uptake on repeated dopamine transporter (DAT) imaging^12^. It was postulated that <50% loss of dopaminergic SN neurons may lead to subtle motor symptoms while >50% loss results in parkinsonism^13^. Accordingly, reduced specific tracer binding ratio (SBR) in the putamen on DAT single-photon emission computed tomography (SPECT) is a sensitive marker of midbrain degeneration which is associated with high risk of imminent conversion to overt synucleinopathy phenotype in RBD^14^ and elderly population^15^. RBD itself is a sleep motor disorder clinically manifesting with jerks, vocalizations and complex motor behaviors during REM sleep alongside with a loss of physiologic muscle atonia. While the dream-enactment behavior apparently waxes and wanes during the time and likely represents only the tip-of-the-iceberg, it has been suggested that phasic and tonic muscle activity recorded using electromyography (EMG) during polysomnography (PSG) may be a quantitative marker reflecting the severity of brainstem neurodegeneration^5^.

Recently, MDS research criteria for prodromal PD have been defined based on the presence of established risk factors and prodromal motor, non-motor, and imaging markers^4^. While the association of these markers with prodromal PD and other synucleinopathies is well documented, the sequence of their onset, rate of progression, and their relationship to SN degeneration are not well explored. Better delineation of these temporal and pathophysiological relationships may improve understanding of the prodromal phase of synucleinopathies.

The aims of this study were (I) to compare prevalence and severity of clinical markers of prodromal synucleinopathy in RBD and control group, and (II) investigate whether these clinical markers, muscle activity during REM sleep, and the probability of prodromal PD according to MDS research criteria, are associated with dopamine transporter binding as a surrogate measure of SN degeneration.

## Methods

### Research participants

A total of 74 (8 female) RBD patients and 39 (7 female) control subjects were included. The diagnosis was confirmed by video-polysomnography according to the International Classification of Sleep Disorders, third edition (ICSD-3)^16^. For inclusion, all patients had to be >49 years, and to be without overt parkinsonism, dementia, severe untreated obstructive sleep apnea (defined as apnea-hypopnea index ≥30) as well as factors indicative of secondary RBD such as narcolepsy, drug-induced RBD (i.e. RBD originating shortly after initiation of antidepressants), or focal brainstem lesions on MRI. Control subjects were recruited from the general community through advertisements. To be eligible for the study, controls had to be >49 years, free of major neurologic disorders, severe obstructive sleep apnea, active oncologic illness, and abuse of psychoactive substances. In all control subjects, RBD was excluded by thorough history and video-polysomnography. All subjects gave written informed consent, ethics approval was obtained from the institutional review board of the General University Hospital in Prague, and the study was carried out in accordance with the recommendations of local ethics guidelines.

### Clinical assessment

Subjects were examined using Movement Disorders Society-Unified Parkinson’s Disease Rating Scale (MDS-UPDRS) by MDS-certified raters. For cognitive evaluation, Montreal Cognitive Assessment (MoCA) was used. Subjects with MoCA score lower than 2.0 SD below the normative mean were classified as mild cognitive impairment (MCI). Mood, anxiety, and sleepiness were assessed using the Beck depression inventory II (BDI-II), State-Trait Anxiety Inventory (STAI) X1/X2, and Epworth sleepiness scale (ESS). Olfaction was investigated using the University of Pennsylvania Smell Identification Test (UPSIT). Color vision was examined using the Farnsworth-Munsell 100 (FM-100) hue test. Autonomic functions were assessed using the Scales for Outcomes in PD-Autonomic (SCOPA-AUT) questionnaire and by the orthostatic test. A drop in systolic/diastolic blood pressure ≥20/10 mm Hg within 3 minutes of standing was considered as positive orthostasis.

### Polysomnography assessment

Video-polysomnography was performed over one night according to to the American Academy of Sleep Medicine (AASM) recommendation with the supplementary recording of superficial EMG of bilateral flexor digitorum superficialis (FDS) muscles. Apnea-hypopnea index (AHI), periodic leg movement index (PLMI), and fraction of REM sleep without atonia (RWA; proportion of REM sleep epochs with at least 50% of the duration of the epoch having a sustained elevated (tonic) muscle activity in the mentalis EMG or excessive transient (phasic) muscle activity in the chin or tibialis anterior EMG) were retrieved. Motor activity during REM sleep was quantified visually according to Sleep Innsbruck Barcelona (SINBAR) criteria for phasic and tonic EMG activity in the mentalis and FDS muscles. All artifacts and increases in EMG tone due to arousals from respiratory events were excluded from the quantitative scoring. Consequently, SINBAR index, that is % of REM sleep with tonic or phasic mentalis EMG activity or phasic FDS EMG activity, was calculated; indexes of tonic and phasic EMG activities assessed acording to SINBAR criteria were also calculated and analyzed separately^17^.

### DAT-SPECT

In 65 RBD patients, DAT-SPECT was performed using the [^123^I]-2-b-carbomethoxy-3b-(4-iodophenyl)-N-(3-fluoropropyl) nortropane ([^123^I]FP-CIT, DaTscan®, GE Healthcare) tracer according to European Association of Nuclear Medicine (EANM) procedure guidelines^18^. Scans were acquired 3 hours after 185 MBq [^123^I]FP-CIT injection on a dual-head camera system (Infinia, GE Healthcare). The acquisition parameters were as follows: rotational radius 13–15 cm, image matrix 128 × 128, angular sampling with 120 projections at 3° interval and 40 seconds per view, zoom 1.3, energy window 159 ± 10% keV. Reconstruction of the raw SPECT projection data was performed using the ordered subset expectation maximization (OSEM) algorithm with 8 iterations and 10 subsets including Chang attenuation correction (μ = 0.11 cm^−1^) and 3D Butterworth post-filtering with FWHM = 8 mm.

**Figure 1:**
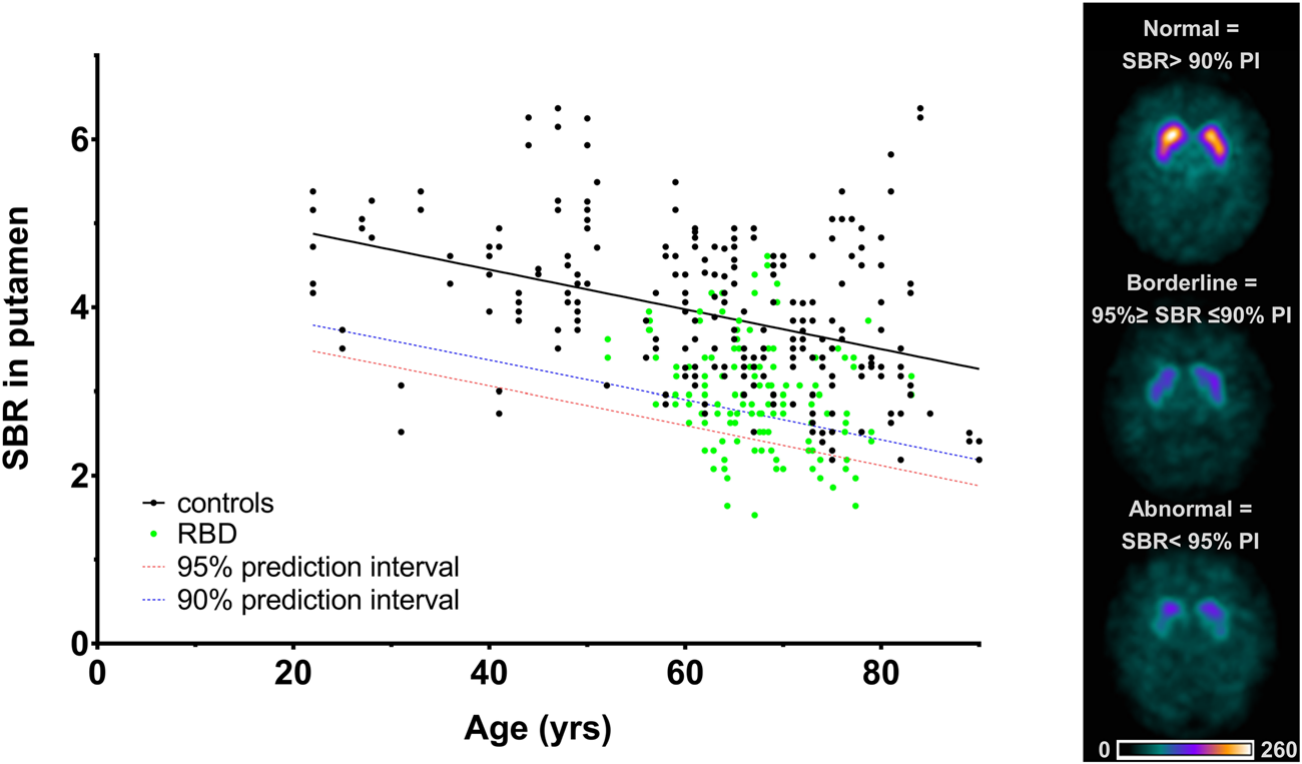
Comparison of putaminal SBR values in controls and RBD patients. On the left, control SBR values in the putamen from both hemispheres are shown in black color together with the 90% (blue) and 95% (red) population prediction intervals. SBR values of RBD patients are shown in green color. On the right, examples of normal, borderline, and abnormal DAT-SPECT images are shown. SBR = specific binding ratio; PI = prediction interval; RBD = REM sleep behavioral disorder.

Automated semi-quantitative analysis was performed using the BasGan V2 software^19^ (https://www.aimn.it/site/page/gds/gds-5) which allows automatic 3D segmentation of the caudate nucleus and putamen based on a high-definition template derived from Talairach space. The calculation of caudate and putaminal binding includes the automatic location of the occipital region of interest (background) and partial volume effect correction. SBR in the caudate nucleus and putamen in each hemisphere are calculated according to the formula [(caudate nucleus or putamen binding–background binding)/background binding]. Reference database with SBR from 97 healthy subjects included in the BasGan V2 software was supplemented with SBR values from 32 internal controls previously examined in our hospital (Supplementary Table 1, Fig. 1). Consequently, linear regression of age and caudate/putamen SBR values in all 129 control subjects was calculated and 90%/95%, one-sided prediction intervals were constructed to define reference range for age-specific normal values (Fig. 1, Supplementary Fig. 2). According to the putaminal SBR from the hemisphere with lower tracer binding, DAT-SPECT scans were stratified as normal (>90% prediction interval), abnormal (<95% prediction interval), and borderline (≤90% and ≥95% prediction interval) (Fig. 1).

### MDS criteria for prodromal PD

The probability of prodromal PD was calculated for each subject using the Bayesian method based on multiplication of likelihood ratios (LR) of assessed markers as described in MDS research criteria for prodromal PD^4^. The following risk factors and prodromal markers were available for the individualized likelihood ratio calculation: sex, pesticide exposure, smoking history, family history of PD, PSG-proven RBD, olfactory dysfunction, constipation, excessive daytime somnolence, symptomatic orthostatic hypotension, erectile dysfunction, urinary dysfunction, diagnosis of depression, subthreshold parkinsonism (MDS-UPDRS-III score >6 excluding postural and action tremor items), and DAT-SPECT in RBD subjects. In the case of ambiguous or missing data, the likelihood ratio of 1 was used. Subjects were regarded positive for prodromal PD when exceeding the recommended probability threshold of 80%.

### Study protocol

Following the screening for inclusion and exclusion criteria, all suspect RBD and control subjects were invited for one-night video-polysomnography. Next day, participants have undergone the protocol consisting of structured interview, MDS-UPDRS, neuropsychologic examination including MoCA, FM-100 hue test, UPSIT, orthostatic test and STAI, BDI-II, and ESS questionnaires. DAT-SPECT was performed within four weeks after polysomnography only in RBD patients who agreed with this examination. From the control group, one subject fulfilled diagnostic criteria for RBD and was moved to the RBD group.

### Statistics

Groupwise comparisons were performed between the RBD and control groups. Distributions of values of continuous variables were tested using the D’Agostino & Pearson normality test. Student t-test or Mann-Whitney U test were used depending on whether data were normally or non-normally distributed. Categorical variables are described as frequency percentages; Chi-square test was used to analyze binary outcome associations. Differences among RBD subgroups were tested depending on normality of the data by the Kruskal-Wallis test or univariate general linear model with the DaTscan result as a fixed factor and age and sex as covariates; post-hoc multiple comparisons tests were applied using the least significant difference method. Correlation-adjusted (using the Dubey/Armitage-Parmar [D/AP] method) Bonferroni procedure was used to correct for multiple comparisons^20^. Partial correlation coefficients with bootstrapped confidence intervals were calculated to examine relationships between mean putaminal SBR and clinical variables controlling for age, sex, and RBD duration. IBM SPSS statistics version 25 (IBM, Armonk, NY, USA) and Graphpad Prism version 6.07 (Graphpad software, San Diego, CA, USA) were used for statistical analysis.

## Results

### Comparison of RBD patients and controls

The characteristics of all participants are shown in Table 1. Compared to control subjects, RBD patients had significantly higher MDS-UPDRS-III, SCOPA-AUT, BDI-II, STAI X1/2, MoCA, FM-100 hue error, and UPSIT scores. Decomposing the total SCOPA-AUT score to individual autonomic region subscores, there was significant difference only in gastrointestinal dysfunction between RBD and controls (Supplementary Table 3). RBD patients also had a higher prevalence of objectively measured orthostasis with the relative risk (RR) = 3.8 (95% confidence interval [CI] 1.6–10.1).

**Table 1:** Comparison of RBD patients and healthy controls.

	RBD	Controls	Uncorrected p-value
**Demography**			
Number (females)	74 (8)	39 (7)	n.a.
Age (years)*	67.5 ± 6.3	65.2 ± 8.2	0.11
Disease duration (years)	6.5 ± 5.8	n.a.	n.a.
Years of schooling*	14.4 ± 3.1	15.1 ± 3.3	0.29
**Motor**			
MDS-UPDRS III^†^	6.4 ± 5.6	3.2 ± 3.2	**0.0005**
**Neuropsychiatric**			
MoCA^†^	23.7 ± 2.8	25.3 ± 2.3	**0.0008**
MCI level I (%)^#^	23.6	10.2	0.09
STAI X1^†^	37.0 ± 10.1	31.2 ± 7.1	**0.001**
STAI X2^†^	39.7 ± 9.3	32.4 ± 7.6	**<0.0001**
BDI II^†^	9.3 ± 7.7	4.6 ± 4.4	**0.0005**
Antidepressant use (%)^#^	20.3	2.6	0.01
**Sensory**			
FM-100 HUE test error score^†^	102.3 ± 78.3	52.4 ± 34.2	**<0.0001**
UPSIT^†^	22.3 ± 7.8	31.3 ± 4.2	**<0.0001**
**Autonomic**			
SCOPA-AUT^†^	11.7 ± 7.6	6.1 ± 3.9	**<0.0001**
Orthostatic test + (%)^#^	41.4	10.8	**0.001**
**Sleep**			
SINBAR index^†^	0.50 ± 0.25	0.06 ± 0.04	**<0.0001**
% RWA^†^	52.6 ± 27.5	2.4 ± 2.5	**<0.0001**
AHI^†^	7.8 ± 8.0	13.8 ± 8.9	**0.0002**
PLMI^†^	35.8 ± 39.1	17.2 ± 25.7	**0.003**
ESS^†^	7.0 ± 3.8	6.0 ± 3.4	0.21

*Values reported as mean ± SD; statistical analysis performed using Student t-test.

^†^Values reported as mean ± SD; statistical analysis performed using Mann-Whitney U test.

^#^Values reported as percent; statistical analysis performed using Chi-square test.

Significant differences after Bonferroni correction are marked with **BOLD** text (for 16 tests with a mean correlation coefficient 0.2 threshold p = 0.0054).

### Associations of clinical features and DAT-SPECT results

Out of the 65 patients who underwent DAT-SPECT, 32 had normal, 16 had borderline and 17 had abnormal findings (Table 2, Supplementary Table 2). RBD patients with abnormal DAT-SPECT had significantly higher MDS-UPDRS-III score and showed a trend towards higher prevalence of objectively measured orthostasis compared to patients with borderline/normal DAT-SPECT. No difference in the total SCOPA-AUT score or cardiovascular dysfunction sub-score between patients with normal and abnormal DAT-SPECT was observed (Supplementary Table 4). Sensitivity analysis performed after merging subgroups with normal and borderline DAT-SPECT into one group returned similar results, i.e. mean MDS-UPDRS-III score and the prevalence of objectively measured orthostasis were significantly higher in patients with abnormal compared to normal DAT-SPECT (Supplementary Table 5).

**Table 2:** Stratification of RBD cohort according to the DaTscan results.

	DaTscan normal	DaTscan borderline	DaTscan abnormal	Uncorrected p-value
**Demography**				
Number (females)	32 (4)	16 (2)	17 (2)	n.a.
Age (yrs)*	67.4 ± 6.7	66.6 ± 6.3	69.3 ± 5.2	0.44
Disease duration (yrs)*	6.5 ± 6.1	5.6 ± 3.8	7.6 ± 6.9	0.61
**DaTSCAN**				
Mean putaminal SBR^†^	3.5 ± 0.5	2.8 ± 0.2	2.3 ± 0.3	**<0.0001** ^**a**^
Mean caudate SBR^†^	4.2 ± 0.5	3.6 ± 0.3	3.2 ± 0.4	**<0.0001** ^**a**^
**Motor**				
MDS-UPDRS III^†^	6.0 ± 4.8	4.0 ± 3.6	10.5 ± 7.5	**0.006** ^**b**^
**Neuropsychiatric**				
MoCA^†^	23.7 ± 2.6	24.3 ± 3.8	22.8 ± 2.8	0.40
MCI level I (%)^#^	21.9	13.3	41.2	0.16
STAI X1^†^	37.0 ± 9.7	35.6 ± 11.6	39.8 ± 10.9	0.46
STAI X2^†^	39.5 ± 9.3	40.1 ± 9.7	42.4 ± 9.9	0.49
BDI II^†^	9.5 ± 7.5	10.9 ± 9.1	9.8 ± 7.7	0.86
Antidepressant use (%)^#^	21.9	25.0	17.6	0.87
**Sensory**				
FM-100 HUE test error score^†^	89.9 ± 51.9	119.6 ± 74.8	90.9 ± 49.4	0.17
UPSIT^†^	23.9 ± 8.2	20.8 ± 8.0	19.8 ± 5.9	0.28
**Autonomic**				
SCOPA-AUT^†^	12.2 ± 6.5	11.1 ± 7.7	13.2 ± 9.7	0.57
Orthostatic test + (%)^#^	24.0	37.5	73.3	0.008
**RBD severity**				
RWA (% of REM sleep)^†^	48.3 ± 28.5	56.5 ± 22.3	58.6 ± 26.1	0.34
SINBAR score^†^	0.44 ± 0.25	0.53 ± 0.19	0.58 ± 0.25	0.13
tonic EMG activity index^†^	0.13 ± 0.13	0.20 ± 0.13	0.25 ± 0.28	0.09
phasic EMG activity index^†^	0.23 ± 0.15	0.24 ± 0.10	0.26 ± 0.15	0.64

*Values reported as mean ± SD; statistical analysis performed using ANOVA test.

^†^Values reported as mean ± SD; statistical analysis performed using univariate general linear model with age and sex as covariates.

^#^Values reported as percent; statistical analysis performed using Chi-square test, ^a^post-hoc tests significant for DAT abnormal vs DAT normal (p = 0.01) and for DAT abnormal vs DAT borderline (p = 0.002), ^b^post-hoc tests significant for DAT abnormal vs DAT normal (p < 0.0001), DAT borderline vs DAT normal (p < 0.0001), and for DAT abnormal vs DAT borderline (p < 0.05).

Significant differences after Bonferroni correction are marked with **BOLD** text (for 15 tests with a mean correlation coefficient 0.22 threshold p = 0.0061).

**Figure 2:**
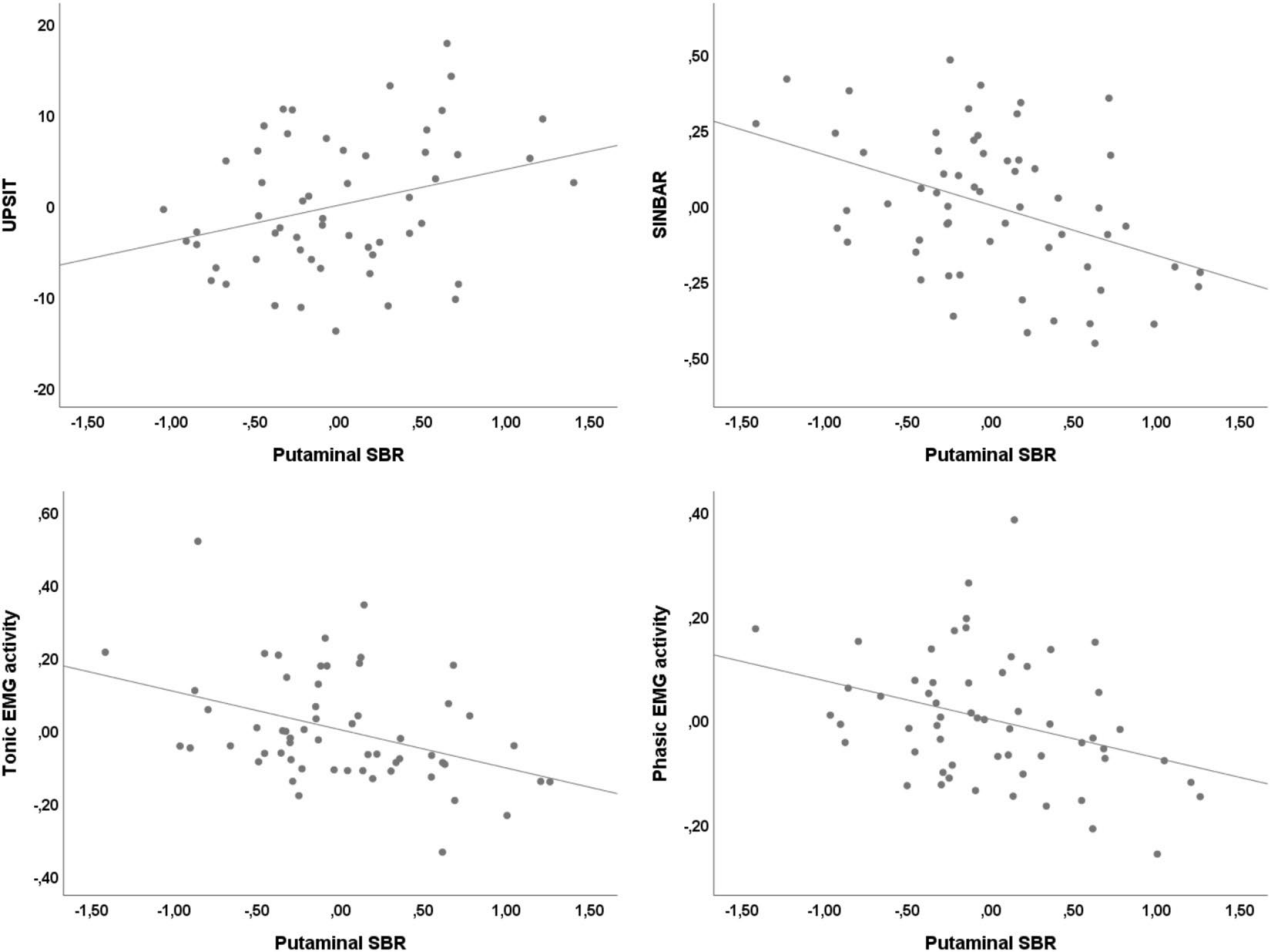
Association of clinical variables with putaminal SBR. Partial regression plots showing the correlation between residual putaminal SBR and selected residual clinical variables; all variables were controlled for age, sex, and RBD duration. SBR = specific binding ratio, UPSIT = University of Pennsylvania Smell Identification Test, SINBAR = Sleep Innsbruck Barcelona group, EMG = electromyography.

Controlling for age, sex, and RBD duration, correlation analysis showed significant positive association between mean putaminal SBR and UPSIT score (r = 0.30, p = 0.03) and negative association between mean putaminal SBR and RWA (r = −0.35, p = 0.009), SINBAR score (r = −0.42, p = 0.001), phasic EMG activity index (r = −0.34, p = 0.01) and tonic EMG activity index (r = −0.39, p = 0.003) (Fig. 2). Correlations between mean putaminal SBR and MDS-UPDRS-III, MoCA, STAI X1/X2, BDI-II, FM-100 HUE test error, and SCOPA-AUT scores were not significant.

### MDS criteria for prodromal PD

None of the control subjects fulfilled the MDS criteria for prodromal PD. On the contrary, 100% RBD patients with abnormal DAT-SPECT fulfilled these criteria compared to 62.5% and 75% RBD patients with normal and borderline DAT-SPECT respectively (Table 3). Prodromal PD probability was significantly larger in the subgroup with abnormal compared to subgroups with normal and borderline DAT-SPECT even when DAT-SPECT item was not included into the calculation. When the RBD item was removed from the probability calculations, 76.5% of patients with abnormal DAT-SPECT still fulfilled criteria for prodromal PD while only one patient from the normal/borderline DAT-SPECT subgroups reached the threshold.

**Table 3:** Prodromal PD criteria.

		Controls	RBD total	RBD DaTscan normal	RBD DaTscan borderline	RBD DaTscan abnormal	P value*
Prodromal PD criteria	posterior probability^†^	0.2% (0.1–0.4)	93.3% (75.2–99.2)	88.5% (51.3–97.1)	89.9% (79.2–97.8)	100% (99.9–100)	**<0.0001** ^**a**^
	% of subjects fulfilling prodromal criteria^*#*^	0	72.0	62.5	75.0	100.0	**0.02**
Prodromal PD criteria (excluding DAT-SPECT item)	posterior probability^†^	n.a.	94.1% (74.6–98.2)	92.2% (74.3–98.1)	93.2% (76.6–97.5)	98.8% (93.1–99.6)	**0.03** ^**b**^
	% of subjects fulfilling prodromal criteria^*#*^	n.a.	74.7	75.0	75.0	88.2	0.52
Prodromal PD criteria (excluding RBD item)	posterior probability^†^	0.3% (0.1–0.7)	30.0% (2.3–47.6)	5.6% (1.5–20.2)	6.5% (1.8–16.3)	96.3% (80.8–99.1)	**<0.0001** ^**a**^
	% of subjects fulfilling prodromal criteria^*#*^	0	18.7	0	6.3	76.5	**<0.0001** ^**a**^

*Statistical analysis was performed on RBD DaTscan normal, borderline, and abnormal subgroups; significant differences are marked with **BOLD** text. Data from controls and RBD total groups are shown for comparison and were not included into the analysis.

^†^Values reported as median (IQR); statistical analysis performed using Kruskal-Wallis test.

^#^Values reported as percent; statistical analysis performed using Chi-square test.

^a^Post-hoc tests significant for DAT abnormal vs DAT normal (p < 0.0001) and for DAT abnormal vs DAT borderline (p < 0.0001).

^b^Post-hoc tests significant for DAT abnormal vs DAT normal (p < 0.01) and for DAT abnormal vs DAT borderline (p < 0.05).

### Effects of antidepressant therapy

Fifteen (20.3%) RBD patients were on antidepressant therapy; sertraline was used in four, citalopram in three, escitalopram in two, and trazodone in two patients, paroxetine, fluoxetine, venlafaxine, bupropion was each used in a single patient. Treatment with serotonin reuptake inhibitors may decrease [^123^I]FP-CIT binding^18^ and increase muscle tone during REM sleep^21^ and thus bias results of this study. We have therefore performed sensitivity analysis comparing RBD subgroups with (AD+) and without (AD−) antidepressant treatment to each other and to healthy controls. Additionally, we have recalculated the comparison of RBD subgroups stratified according to DAT-SPECT status excluding the AD+ RBD subgroup. In comparison to healthy controls, significant differences in clinical features was the same for AD+ and AD− RBD subgroups (Supplementary Table 6). Compared to AD− subgroup, AD+ subgroup had significantly higher STAI-X1 (p = 0.001), STAI-X2 (p = 0.02), BDI-II (p = 0.003), and UPSIT (p = 0.02) scores. The pattern of differences between RBD subgroups with normal, borderline, and abnormal DAT-SPECT were not altered after excluding the AD+ subgroup from the analysis, i.e. trend towards higher mean MDS-UPDRS-III score and higher prevalence of objectively measured orthostasis was shown in patients with abnormal compared to normal/borderline DAT-SPECT (Supplementary Table 7).

## Discussion

In this study, we have cross-sectionally examined a large cohort of RBD patients with presumably different stages of synucleinopathy progression to analyze associations between the degree of SN degeneration and other clinical features of prodromal synucleinopathy. We have demonstrated worse motor function and more prevalent orthostasis in RBD subgroup with abnormal compared to normal DAT-SPECT. Furthermore, we have shown that dopamine transporter binding is associated with olfactory dysfunction and severity of EMG activity during REM sleep.

High prevalence of motor and non-motor symptoms indicative of widespread impairment of central and peripheral nervous system found in this RBD cohort is in agreement with previous research^22,23^. Accordingly, other studies showed that the presence of RBD identifies more severe synucleinopathy subtype, also referred to as “diffuse malignant phenotype” which is characterized by a higher prevalence of cognitive impairment, dysautonomia, and gait abnormalities^24^.

MDS-UPDRS-III score was increased only in the RBD subgroup with abnormal compared to subgroups with normal/borderline DAT-SPECT. This, along with the lack of linear correlation between MDS-UPDRS-III score and putaminal SBR, supports the threshold effect in the influence of SN dopaminergic damage on motor function^13^. Here it is important to note that raters were not aware of the DAT-SPECT results when examining MDS-UPDRS. In line with our results, a significant association between low striatal SBR, motor dysfunction, and smell loss was documented in a community cohort with a high risk of developing PD^25^. Relative risk of abnormal striatal SBR was found to be 12.4 for hyposmic compared to normosmic elderly subjects which further supports the link between SN dopaminergic degeneration and olfactory loss^26^. On the other hand, no differences in MDS-UPDRS score between RBD patients with normal and abnormal DAT-SPECT were found in a recent study^27^. These disparate findings may be caused by more stringent criteria for abnormal DAT-SPECT used in our study.

The prevalence of orthostasis was apparently increased in the subgroup with abnormal DAT-SPECT compared to the subgroup with normal DAT-SPECT. Orthostatic hypotension is a frequent symptom of synucleinopathies; it is presumably caused by cardiac sympathetic denervation along with diffusely decreased noradrenergic innervation throughout the body, and degeneration of the solitary nucleus in the brainstem^28,29^. Presence of objectively assessed orthostatic hypotension in the elderly may be a stronger marker of prodromal synucleinopathy than previously thought as documented by LR of 8.3 found in a recent study^30^. On the contrary, we found no association between the total SCOPA-AUT score or cardiovascular dysfunction subscore and DAT-SPECT binding. While blood pressure drop in the orthostatic test is an objective marker, self-reported symptoms in the SCOPA-AUT questionnaire may not be related to autonomic dysfunction. Additionally, the degree of degeneration in the autonomic system may not linearly translate to clinical symptoms. In any case, objective orthostatic test appears to be superior to questionnaire-based assessment of orthostatic dysfunction in the stratification of severity in synucleinopathies.

We observed no relation between degeneration of SN and cognitive dysfunction, color discrimination, and questionnaire-based assessment of depression and anxiety. Cognitive and color discrimination impairment are both likely caused by neurodegeneration of cortical areas in PD and RBD^31,32^. Lack of correlation between DAT-SPECT binding and severity of cognitive symptoms could indicate that neurodegeneration in RBD advances concomitantly but independently in the SN and cortical regions. This has been already suggested based on mathematical modelling of PD and DLB incidences in a meta-analysis of longitudinal RBD studies^33^.

Quantitative assessment showed a significant association between markers of REM sleep motor activity and SN degeneration; stronger association was observed for tonic as compared to phasic EMG muscle activity. These findings confirm and extend results of previous studies indicating an association between increased EMG activity during REM sleep and decreased DAT-SPECT binding in groups consisting of patients with RWA, RBD, and PD^34^. Our data show that EMG muscle activity is positively associated with the severity of SN degeneration even in a homogenous cohort of RBD patients. Accordingly, high EMG (particularly tonic) muscle activity in RBD was previously associated with early phenoconversion to PD^35,36^. It has been suggested that abnormal muscle activity during REM sleep likely reflects degeneration of nucleus subcoeruleus and/or ventromedial medulla which is anatomically proximal to substantia nigra^37^. It is thus theoretically possible that in RBD alpha-synuclein pathology spreads to adjacent SN once the subcoeruleus nucleus is affected by the alpha-synuclein to a certain degree. Alternatively, subclinical compromise of dopaminergic neurotransmission may exert an additive effect on pontine or medullary structures involved in REM sleep regulation and thus accentuate impaired muscle tone control during REM sleep.

The prevalence 74.7% of prodromal PD diagnosed according to MDS research criteria (excluding DAT-SPECT item) in our study is comparable to other studies which found 60.3%^38^ and 73.7%^22^ prevalence in RBD subjects. The high prevalence of prodromal PD in RBD patients reflects the highest LR of PSG-proven RBD (LR = 130) among prodromal symptoms. Indeed, when RBD status was excluded from the calculation, only 18.7% of patients fulfilled these criteria. The value 18.7% in our study is seemingly in contrast with 6.6%^38^ and 45%^39^ prevalence reported in previous studies. However, the former study did not include DAT imaging and the latter study included a cohort with long-standing disease and a high proportion of subjects with abnormal DAT-SPECT, which likely explains the differences. MDS prodromal criteria were validated in an RBD cohort and showed sensitivity/specificity of 81.3%/67.0% for phenoconversion within four years of follow-up. Moreover, higher conversion rates were observed with higher baseline probability of prodromal PD^38^. The pros and cons of using MDS prodromal PD criteria in RBD patients have been extensively discussed^40^. Our results add several points to this ongoing discussion: i) prodromal PD probability is higher in patients with abnormal DAT-SPECT (even when DAT-SPECT item is excluded from the calculation) suggesting more advanced neurodegeneration in this subgroup and indicating that calculation of prodromal PD probability may be helpful in stratification of the RBD cohort, ii) when RBD status is excluded from the calculation, positive DAT-SPECT status is the crucial factor for fulfilling MDS prodromal criteria. MDS prodromal criteria including DAT-SPECT and excluding RBD status may thus theoretically allow for accurate estimation of phenoconversion risk in RBD patients.

In comparison to healthy controls, RBD AD+ subgroup showed a similar pattern of prodromal markers as RBD AD− subgroup. This is in line with the previous study which documented the consistent presence of neurodegenerative signs but lower risk for phenoconversion in RBD patients on antidepressant treatment^41^. RBD AD+ subgroup had generally milder point estimates of examined clinical scores compared to RBD AD− subgroup, but higher UPSIT score in the AD+ subgroup was the only significant between-group difference. A similar finding was recently reported in another RBD cohort^22^. The difference in olfactory function between AD+ and AD− RBD subgroups is striking and should be examined in future prospective studies. Interestingly, beneficial effect of antidepressants on olfactory function was described in patients with major depression^42^ and this potential influence of antidepressants on olfaction is worth further investigation also in RBD.

Some limitations should be noted. First, cross-sectional design does not allow calculations of phenoconversion risk and assessment of dynamic changes in severity of symptoms with relation to DAT-SPECT binding. This will be targeted by future studies on this cohort. Second, although there are no other known causes of idiopathic RBD beside alpha-synucleinopathy and we carefully included only patients without signs of secondary RBD, the possibility that subjects without synucleinopathy were included (e.g., by a diagnostic error of RBD and other REM parasomnias) cannot be ruled out. This would have biased our analyses and inferences about relations of synucleinopathy symptoms. Nevertheless, it is unlikely that a substantial number of misdiagnosed patients were included to bias main outcomes of this study. Third, antidepressants were not discontinued before DAT-SPECT exam and could have thus altered DAT binding. However, our results of the DAT-SPECT stratification analysis persisted after excluding the AD+ subgroup arguing against the profound bias of antidepressants. Lastly, MoCA score, chosen as a simple and widely used marker of cognitive status, has only limited sensitivity for assessment of overall cognitive abilities and defining MCI status.

In conclusion, we have confirmed that RBD is associated with hyposmia, autonomic dysfunction, anxiety, depression, cognitive impairment, and mild motor symptoms indicating diffuse alpha-synuclein pathology. SN degeneration is associated with motor impairment, olfactory dysfunction, orthostatic hypotension, severity of EMG activity during REM sleep, and higher probability of prodromal PD according to MDS criteria. No associations were observed for DAT-SPECT binding and cognitive impairment and color sensitivity. These results suggest that, in RBD, neurodegeneration advances simultaneously in susceptible brainstem nuclei and olfactory pathway while concomitant cortical degeneration advances independently.

## Supplementary information

Supplementry material

## Data Availability

The datasets generated during and/or analysed during the current study are available from the corresponding author on reasonable request.
