# Supplementary material for: Relations of non-motor symptoms and dopamine transporter binding in REM sleep behavior disorder
Source: Sci Rep. 2019 Oct 29;9:15463. doi: 10.1038/s41598-019-51710-y (PMC6820530; doi:10.1038/s41598-019-51710-y)
Supplement: Supplementary file 1 — Supplementry material [file 41598_2019_51710_MOESM1_ESM.docx]

**Supplementary material**

**Relations of non-motor symptoms and** **dopamine transporter binding in REM sleep behavior disorder**

Petr Dušek^1^, Veronika Ibarburu Lorenzo y Losada^1^, Ondrej Bezdicek^1^, Irene Dall’Antonia^1^, Simona Dostálová^1^, Petra Kovalská^1^, Radim Krupička^3^, Jiří Nepožitek^1^, Tomáš Nikolai^1^, Michal Novotný^4^, Pavla Peřinová^1^, Jan Rusz^1,4^, Tereza Serranová^1^, Tereza Tykalová^4^, Olga Ulmanová^1^, Zuzana Mecková^2^, Václav Ptáčník^2^, Jiří Trnka^2^, David Zogala^2^, Evžen Růžička^1^, Karel Šonka^1^

^1^Department of Neurology and Center of Clinical Neuroscience, First Faculty of Medicine, Charles University and General University Hospital in Prague, Czech Republic

^2^Institute of Nuclear Medicine, First Faculty of Medicine, Charles University and General University Hospital in Prague, Czech Republic

^3^Department of Biomedical Informatics, Faculty of Biomedical Engineering, Czech Technical University in Prague, Kladno, Czech Republic

^4^Department of Circuit Theory, Faculty of Electrical Engineering, Czech Technical University in Prague, Prague, Czech Republic

1. **Comparison of DAT-SPECT binding values in control subjects from the Basgan_v2 software and internal controls from General University Hospital in Prague**

In order to confirm that specific tracer binding ratio (SBR) values from the Basgan_v2 software are valid for subjects examined at the General University Hospital (GUH) in Prague, we compared these Basgan_v2 SBR values to values from internal controls examined earlier (2005 - 2008) at the GUH in Prague. DAT-SPECT in these diseased control subjects was performed for diagnostic purposes; final diagnoses were essential tremor, functional disorder, drug-induced parkinsonism, or dystonia. SBR values were compared for the caudate nucleus and putamen from each hemisphere separately using the univariate general linear model with the source of the data (Basgan_v2 vs. GUH in Prague) as a fixed factor and age and sex as covariates. No significant differences between control SBR values provided with the Basgan_v2 software and SBR values measured at GUH in Prague were observed (Supplementary Table 1, Supplementary Figure 1). Therefore, SBRs from the Basgan_v2 software can be used as reference values for subjects examined at GUH in Prague.

| **Supplementary Table 1. comparison of SBR values in control subjects** | | | |
| --- | --- | --- | --- |
|  | **Basgan_v2** | **GUH Prg** | **P value** |
| Number of subjects (females) | 97 (65) | 32 (18) | n.a. |
| Age* | 65.2 ± 15.3 | 57.8 ± 14.2 | **0.02** |
| Left Caudate nucleus SBR† | 4.72 ± 1.10 | 4.69 ± 0.78 | 0.32 |
| Right Caudate nucleus SBR† | 4.74 ± 1.09 | 4.69 ± 0.70 | 0.25 |
| Left Putamen SBR† | 3.93 ± 0.97 | 3.80 ± 0.65 | 0.11 |
| Right Putamen SBR† | 3.94 ± 0.98 | 3.74 ± 0.69 | 0.07 |
| ** values reported as mean ± SD; statistical analysis performed using the Student t-test*  † *values reported as mean ± SD; statistical analysis performed using the univariate general linear model with age and sex as covariates*  *Abbreviations: SBR = specific binding ratio; GUH Prg = General University Hospital in Prague* | | | |


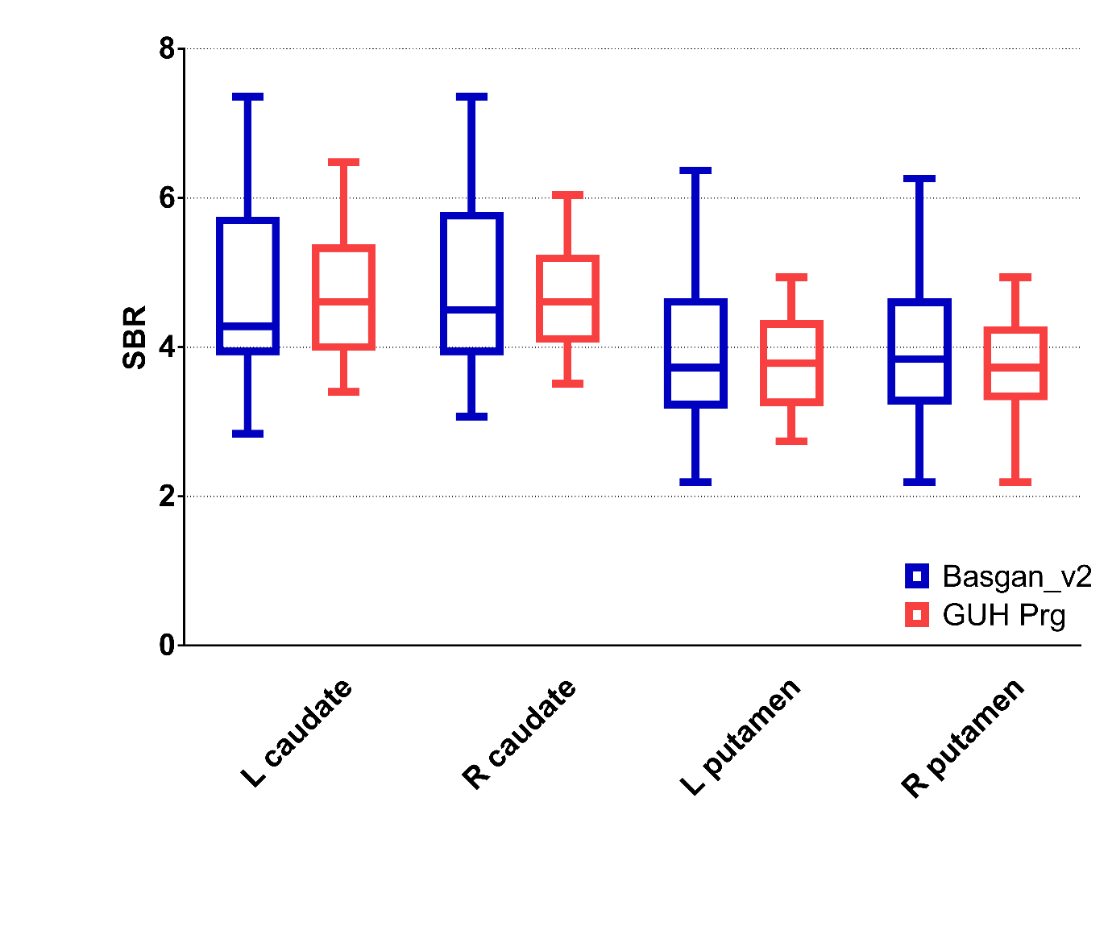


***Supplementary Figure 1. Comparison of SBR values in control subjects****Boxplot graph showing comparison of specific binding ratio (SBR) values in control subjects from the Basgan_v2 software and internal control subjects from the General University Hospital in Prague (GUH Prg).*

1. **Construction of population prediction intervals for DAT specific tracer binding ratios**

SBRs for both hemispheres from all control subjects from the Basgan_v2 software and GUH Prg were combined yielding total of 258 reference values for the caudate nucleus as well as for putamen. Linear regression of age and SBR was calculated for the caudate nucleus and putamen, and one-sided 90% and 95% population prediction intervals were constructed. Next, RBD patients were stratified according to the lower putaminal SBR from both hemispheres. Subjects with values below the 95% prediction interval were considered to have abnormal DAT SPECT and subjects with values above the 90% prediction interval were considered to have normal DAT SPECT. Values between the 90% and 95% prediction intervals were considered as borderline DAT SPECT.


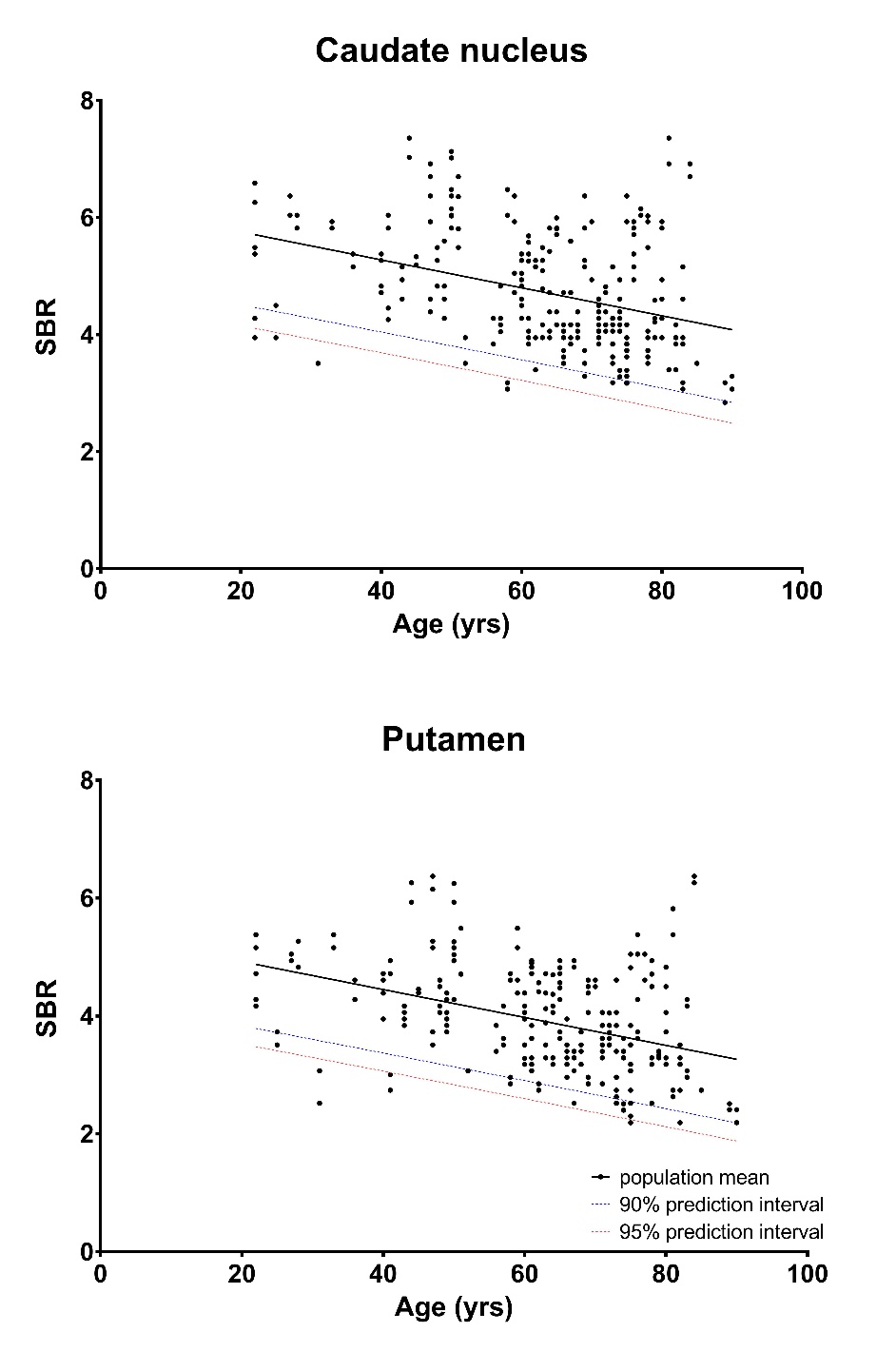


***Supplementary Figure 2. Construction of population prediction intervals****Control SBR values in the caudate nucleus and putamen from both hemispheres sourced from Basgan_v2 and GUH Prg plotted against age. The one-sided 90% and 95% prediction intervals are shown in blue and red color respectively; 10% and 5% of SBR values from healthy population respectively are expected to be below these cut-off intervals.*

1. **Comparison of DAT-SPECT results in RBD patients and internal control subjects**

For comparison of SBR values between RBD patients and control subjects, values from local controls older than 49 years (to keep age-matching between groups) were used. RBD patients had significantly lower SBR values in the caudate nucleus and putamen from both hemispheres (Supplementary Table 2).

| **Supplementary Table 2. comparison of SBR values in RBD and internal control subjects** | | | |
| --- | --- | --- | --- |
|  | **RBD patients** | **GUH Prg controls** | **Uncorrected p-value** |
| Number of subjects (females) | 65 (8) | 23 (12) | n.a. |
| Age* | 67.7 ± 6.3 | 64.5 ± 9.5 | 0.15 |
| Left Caudate nucleus SBR† | 3.79 ± 0.64 | 4.49 ± 0.79 | **0.007** |
| Right Caudate nucleus SBR† | 3.74 ± 0.62 | 4.51 ± 0.72 | **0.001** |
| Left Putamen SBR† | 3.01 ± 0.62 | 3.60 ± 0.63 | **0.012** |
| Right Putamen SBR† | 2.93 ± 0.67 | 3.51 ± 0.6 | **0.022** |
| ** values reported as mean ± SD; statistical analysis performed using the Student t-test*  † *values reported as mean ± SD; statistical analysis performed using the univariate general linear model with age and sex as covariates*  *Abbreviations: SBR = specific binding ratio; GUH Prg = General University Hospital in Prague*  ***significant*** *differences after Bonferroni correction are marked with* ***BOLD*** *text (for 4 tests with a mean correlation coefficient 0.844 threshold p = 0.04)* | | | |

1. **Decomposing the SCOPA-AUT total score into autonomic region subscores**

| **Supplementary Table 3. Comparison of SCOPA-AUT region subscores in RBD and control subjects** | | | |
| --- | --- | --- | --- |
|  | **RBD** | **Controls** | **Uncorrected p-value** |
| Gastrointestinal dysfunction* | 2.9 ± 2.7 | 0.9 ± 1.1 | **<0.0001** |
| Urinary dysfunction * | 4.2 ± 3.2 | 2.7 ± 1.9 | 0.02 |
| Cardiovascular dysfunction * | 0.7 ± 1.2 | 0.3 ± 0.5 | 0.06 |
| Pupillomotor dysfunction * | 0.2 ± 0.5 | 0.3 ± 0.4 | 0.60 |
| Thermoregulatory dysfunction * | 2.1 ± 2.0 | 1.2 ± 1.2 | 0.06 |
| Sexual dysfunction * | 1.6 ± 1.7 | 0.8 ± 1.3 | 0.04 |
| ** values reported as mean ± SD; statistical analysis performed using Mann-Whitney U test*  ***significant*** *differences after Bonferroni correction are marked with* ***BOLD*** *text (for 6 tests with a mean correlation coefficient 0.315 threshold p = 0.015)* | | | |

| **Supplementary Table 4. Comparison of SCOPA-AUT region subscores in RBD patients stratified according to DAT-SPECT** | | | | |
| --- | --- | --- | --- | --- |
|  | **DaTscan normal** | **DaTscan borderline** | **DaTscan abnormal** | **Uncorrected p-value** |
| Gastrointestinal dysfunction * | 3.0 ± 2.4 | 2.4 ± 2.2 | 3.9 ± 3.8 | 0.42 |
| Urinary dysfunction * | 3.9 ± 2.9 | 4.4 ± 3.0 | 5.3 ± 3.9 | 0.50 |
| Cardiovascular dysfunction * | 0.8 ± 1.3 | 1.1 ± 1.7 | 0.4 ± 0.8 | 0.49 |
| Pupillomotor dysfunction * | 0.4 ± 0.7 | 0.1 ± 0.3 | 0.2 ± 0.4 | 0.23 |
| Thermoregulatory dysfunction * | 2.7 ± 2.1 | 1.8 ± 1.6 | 1.7 ± 2.0 | 0.12 |
| Sexual dysfunction * | 1.5 ± 1.7 | 1.5 ± 1.5 | 1.7 ± 2.0 | 0.98 |
| ** values reported as mean ± SD; statistical analysis performed using Kruskal-Wallis test* | | | | |

1. **Comparison of clinical markers in RBD patients with normal and abnormal DaTscan**

| **Supplementary Table 5. Comparison of RBD patients with normal and abnormal DaTscan results** | | | |
| --- | --- | --- | --- |
|  | **DaTscan normal** | **DaTscan abnormal** | **Uncorrected**  **p-value** |
| Number (females) | 48 (6) | 17 (2) | n.a. |
| Age (yrs)* | 67.1 ± 6.5 | 69.3 ± 5.2 | 0.23 |
| Disease duration (yrs)* | 6.1 ± 5.4 | 7.6 ± 6.9 | 0.44 |
| MDS-UPDRS III† | 5.4 ± 4.5 | 10.5 ± 7.5 | **0.003** |
| MoCA† | 23.9 ± 3.0 | 22.8 ± 2.8 | 0.21 |
| MCI level I (%)^#^ | 19.2 | 41.2 | 0.07 |
| STAI X1† | 36.6 ± 10.2 | 39.8 ± 10.9 | 0.25 |
| STAI X2† | 39.7 ± 9.4 | 42.4 ± 9.9 | 0.23 |
| BDI II† | 10.0 ± 8.0 | 9.8 ± 7.7 | 0.97 |
| Antidepressant use (%)^#^ | 22.9 | 17.6 | 0.65 |
| FM-100 HUE test error score† | 99.8 ± 61.3 | 90.9 ± 49.4 | 0.49 |
| UPSIT† | 23.0 ± 8.2 | 19.8 ± 5.9 | 0.24 |
| SCOPA-AUT† | 11.9 ± 6.9 | 13.2 ± 9.7 | 0.37 |
| Orthostatic test + (%)^#^ | 29.3 | 73.3 | **0.003** |
| RWA (% of REM sleep) † | 51.5 ± 26.6 | 60.0 ± 25.9 | 0.28 |
| SINBAR score† | 0.48 ± 0.23 | 0.59 ± 0.25 | 0.10 |
| tonic EMG activity index† | 0.15 ± 0.13 | 0.22 ± 0.24 | 0.20 |
| phasic EMG activity index† | 0.24 ± 0.13 | 0.24 ± 0.14 | 0.96 |
| ** values reported as mean ± SD; statistical analysis performed using ANOVA test*  † *values reported as mean ± SD; statistical analysis performed using univariate general linear model with age and sex as covariates*  *^#^ values reported as percent; statistical analysis performed using Chi-square test*  ***significant*** *differences after Bonferroni correction are marked with* ***BOLD*** *text (for 15 tests with a mean correlation coefficient 0.22 threshold p = 0.0061)* | | | |

1. **Comparison of RBD subgroups with and without antidepressant therapy**

| **Supplementary Table 6. Comparison of RBD AD+ and AD- subgroups and healthy controls** | | | | | |
| --- | --- | --- | --- | --- | --- |
|  | **Controls (A)** | **RBD AD+ (B)** | **RBD AD- (C)** | **Uncorrected p-value** | **Post-hoc tests** |
| **Demography** | | | | | |
| Number (females) | 39 (7) | 15 (1) | 59 (8) | n.a. | n.a. |
| Age (years)‡ | 65.2 ± 8.2 | 66.3 ± 6.3 | 67.8 ± 6.3 | 0.22 | n.a. |
| Disease duration (years) ‡‡ | n.a. | 5.1 ± 3.2 | 6.8 ± 6.2 | 0.31 | n.a. |
| Years of schooling‡ | 15.1 ± 3.3 | 14.3 ± 3.0 | 14.4 ± 3.2 | 0.57 | n.a. |
| **Motor** | | | | | |
| MDS-UPDRS III† | 3.2 ± 3.2 | 6.7 ± 7.0 | 6.3 ± 5.3 | 0.009 | A<B*, A<C** |
| **Neuropsychiatric** | | | | | |
| MoCA† | 25.3 ± 2.3 | 23.7 ± 2.3 | 23.7 ± 2.9 | 0.01 | A>B*, A>C** |
| MCI level 1 (%)^#^ | 10.2 | 33.3 | 21.1 | 0.13 | n.a. |
| STAI X1† | 31.2 ± 7.1 | 43.9 ± 11.5 | 35.2 ± 8.9 | **<0.0001** | **A<B***, A<C*, B>C***** |
| STAI X2† | 32.4 ±7.6 | 44.7 ± 11.8 | 38.4 ± 8.1 | **<0.0001** | **A<B***, A<C***, B>C*** |
| BDI II† | 4.6 ± 4.4 | 13.7 ± 9.4 | 8.2 ± 6.9 | **<0.0001** | **A<B***, A<C*, B>C**** |
| **Sensory** | | | | | |
| HUE test† | 52.4 ± 34.2 | 77.5 ± 61.1 | 108.6 ± 81.4 | **0.001** | **A<C***** |
| UPSIT† | 31.3 ± 4.2 | 26.1 ± 6.9 | 21.2 ± 7.7 | **<0.0001** | **A>B*, A>C***, B>C*** |
| **Autonomic** | | | | | |
| SCOPA-AUT† | 6.1 ± 3.9 | 13.1 ± 8.8 | 11.3 ± 7.3 | **0.0002** | **A<B***, A<C***** |
| Orthostatic test + (%)^#^ | 10.8 | 23.1 | 42.1 | **0.004** | **A<C**** |
| **Sleep** | | | | | |
| SINBAR index† | 0.06 ± 0.04 | 0.41 ± 0.23 | 0.51 ± 0.24 | **<0.0001** | **A<B***, A<C***** |
| % RWA† | 2.4 ± 2.5 | 45.1 ± 25.8 | 53.5 ± 27.4 | **<0.0001** | **A<B***, A<C***** |
| AHI† | 13.8 ± 8.9 | 10.8 ± 8.8 | 7.0 ± 7.7 | **0.0003** | **A>C***** |
| PLMI† | 17.2 ± 25.7 | 45.1 ± 47.0 | 33.5 ± 37.1 | 0.07 | n.a. |
| ESS† | 6.0 ± 3.4 | 8.1 ± 4.6 | 6.7 ± 3.6 | 0.15 | n.a. |
| **DAT SPECT and prodromal criteria** | | | | | |
| Mean putaminal SBR‡‡ | n.a. | 3.2 ± 0.7 | 2.9 ± 0.6 | 0.15 | n.a. |
| Prodromal PD probability‡‡‡ | 0.2% (0.1-0.4%) | 91.0% (56.0 – 100.0%) | 94.0% (81.3 – 99.0%) | 0.65 | n.a. |
| patients fulfilling prodromal criteria (%)^#^ | 0 | 60.0 | 74.6 | **<0.0001** | **A<B***, A<C***** |
| ‡ *values reported as mean ± SD; statistical analysis performed using ANOVA*  ‡‡ *values reported as mean ± SD; statistical analysis performed using Student t-test*  ‡‡‡ *values reported as median (IQR); statistical analysis performed only between AD+ and AD- subgroups using Mann-Whitney U test*  † *values reported as mean ± SD; statistical analysis performed using univariate general linear model with age and sex as covariates, post-hoc test performed using least square difference (LSD) method*  *^#^ values reported as percent; statistical analysis performed using Chi-square test*  ** <0.05, ** <0.01, *** <0.001*  ***significant*** *differences after Bonferroni correction are marked with* ***BOLD*** *text (for 18 tests with a mean correlation coefficient 0.21 threshold p = 0.0051)* | | | | | |

| **Supplementary Table 7. Stratification of RBD cohort according to DaTscan results excluding patients on antidepressant therapy** | | | | |
| --- | --- | --- | --- | --- |
|  | **DaTscan normal** | **DaTscan borderline** | **DaTscan abnormal** | **Uncorrected p-value** |
| **Demography** | | | | |
| Number (females) | 25 (4) | 12 (2) | 14 (2) | n.a. |
| Age (yrs)* | 68.0 ± 6.3 | 66.1 ± 7.0 | 69.3 ± 5.5 | 0.43 |
| Disease duration (yrs)* | 7.2 ± 6.6 | 6.0 ± 4.1 | 7.8 ± 7.6 | 0.78 |
| **Motor** | | | | |
| MDS-UPDRS III† | 6.0 ± 4.6 | 3.8 ± 2.9 | 9.9 ± 7.1 | 0.03 |
| **Neuropsychiatric** | | | | |
| MoCA† | 23.6 ± 2.8 | 24.5 ± 4.3 | 23.1 ± 2.4 | 0.54 |
| MCI level 1 (%)^#^ | 20.0 | 9.1 | 35.7 | 0.26 |
| STAI X1† | 34.6 ± 8.3 | 34.2 ± 11.3 | 35.4 ± 9.2 | 0.49 |
| STAI X2† | 37.5 ± 8.1 | 40.3 ± 8.5 | 40.2 ± 8.9 | 0.48 |
| BDI II† | 7.8 ± 6.6 | 10.6 ± 9.3 | 8.3 ± 5.4 | 0.53 |
| **Sensory** | | | | |
| HUE test† | 89.8 ± 49.1 | 130.0 ± 74.2 | 98.9 ± 50.1 | 0.08 |
| UPSIT† | 22.3 ± 8.1 | 20.1 ± 8.7 | 19.5 ± 5.9 | 0.63 |
| **Autonomic** | | | | |
| SCOPA-AUT† | 11.8 ± 5.4 | 10.5 ± 7.9 | 12.6 ± 9.9 | 0.63 |
| Orthostatic test + (%)^#^ | 29.4 | 41.7 | 76.9 | 0.03 |
| **RBD severity** | | | | |
| RWA (% of REM sleep) † | 49.3 ± 29.3 | 57.7 ± 22.6 | 62.9 ± 24.5 | 0.28 |
| SINBAR score† | 0.46 ± 0.25 | 0.54 ± 0.22 | 0.62 ± 0.25 | 0.13 |
| tonic EMG activity index† | 0.13 ± 0.13 | 0.22 ± 0.22 | 0.23 ± 0.24 | 0.19 |
| phasic EMG activity index† | 0.25 ± 0.16 | 0.25 ± 0.11 | 0.25 ± 0.14 | 0.99 |
| ** values reported as mean ± SD; statistical analysis performed using ANOVA test*  † *values reported as mean ± SD; statistical analysis performed using univariate general linear model with age and sex as covariates*  *^#^ values reported as percent; statistical analysis performed using Chi-square test*  *no significant differences were observed after Bonferroni correction (for 15 tests with a mean correlation coefficient 0.22 threshold p = 0.0061)* | | | | |
